# Reference-Free Quantitative Mass Spectrometry Enables Sequencing of Resist Copolymers and Reveals Sequence-Dependent Deprotection Sensitivity

**DOI:** 10.1021/acs.macromol.5c03032

**Published:** 2026-01-19

**Authors:** Yusuke Hibi, Yasuyuki Nakamura, Shiho Uesaka, Masanobu Naito

**Affiliations:** † Data-Driven Polymer Design Group, Research Center for Macromolecules and Biomaterials, 52747 National Institute for Materials Science (NIMS); 1-2-1, Sengen, Tsukuba, Ibaraki 305-0047, Japan

## Abstract

The influence of monomer sequence in resist polymers on line-edge roughness (LER) has long remained elusive in semiconductor lithography. Although the arrangement of degradable and nondegradable monomers should affect polymer solubility in developer solutions, the lack of sequencing methods has prevented analysis of sequence–LER correlations. Here, we present a sequencing approach for resist polymers using pyrolysis mass spectrometry (pyrolysis-MS), which quantifies short-sequence frequencies from pyrolyzed oligomer fragments. Methacrylate-based resist polymers, however, undergo depolymerization and side chain cleavage, generating fragments too small to retain sequence information. Nevertheless, we found these instabilities themselves are sequence-dependent, as shown by computational modeling, encoding sequence information in decomposition temperature profiles. By exploiting both mass- and temperature-domains, our strategy enables sequencing of resist copolymers previously considered inaccessible. Moreover, sequence-dependent side chain instabilities imply that resist responsiveness in deprotection processes may also depend on sequence. The proposed sequencer offers a path to unravel the long-standing sequence–LER relationship.

## Graphical abstract

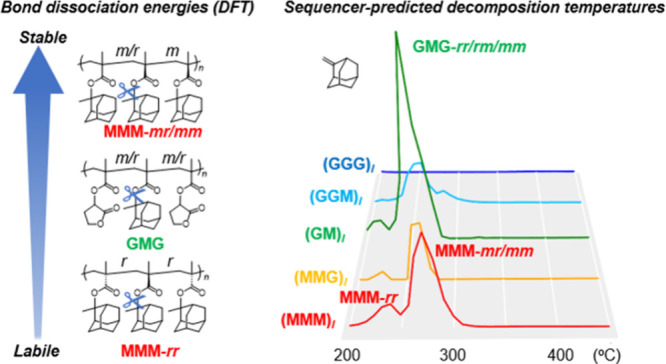

## Introduction

Chemically amplified resists have been the mainstream in current semiconductor manufacturing process. These resist polymers are composed of monomers that are susceptible or stable to acid-catalyzed side chain decomposition.^1^ Upon exposure to light, protons generated from the photoacid generators catalyze the side chain cleavage, converting the decomposable monomeric units into methacrylic acids or phenols. This makes the light-exposed regions soluble in alkaline developer solutions, providing patterning templates in subsequent dry etching process. Ideally, only the areas exposed to light should be dissolved sharply and cleanly, but in practice, incomplete dissolution and acid diffusion into unexposed areas makes the pattern boundaries fuzzy.^2,3^ As this can lead to circuit errors, line-edge roughness (LER) is a critical parameter of resist polymers.

From a polymer science perspective, LER has been attributed to the structural heterogeneities in molecular weight and monomer sequence distribution of the resist polymer.^1,4^ However, it was reported that narrowing molecular weight distribution did not improve the LER,^5^ leading to the hypothesis that sequence distribution might be the determining factor. Indeed, in polypeptoid-based resists, strictly defined sequences have been synthesized and the impact of sequence on LER has been investigated.^6,7^ By contrast, for the vinyl polymers that have long been employed in industrial resists, precise sequence control is inherently difficult, and even quantitative evaluation of the sequence distribution has remained challenging.^8^ Polymer sequence has conventionally been analyzed using nuclear magnetic resonance (NMR) spectroscopy, in which sequence-sensitive peak shifts are exploited to assign triad sequences, as demonstrated in several previous studies.^9,10^ However, as the chemical structure of resist polymers has become more complex to meet various demands, such as dry etching resistance, substrate wetting properties, and acid-catalyzed thermal degradability, their NMR spectra have become too busy with many overlapped peaks, making sequence analysis nearly impossible.^11,12^ The problem is further compounded by the fact that many of these monomers share a common backbone structure, resulting in no sequence-sensitive peaks appearing in the NMR spectra. However, as patterning pitches approach just a few nanometers, equivalent to the scale of a few monomers, sequence distribution becomes more dominant in resist process. For example, if acid-catalyzed decomposable monomers are sparsely distributed in the light-exposed regions, the failure of the resist polymers to gain sufficient solubility is intuitively understandable.^4^ The rapid development in semiconductor industry imposes a high demand on new sequencing technologies.

As an alternative characterization method, mass spectrometry (MS) has recently attracted increasing attention owing to advances in high-resolution instrumentation and large-scale data analysis techniques.^13–18^ Nevertheless, quantitatively determining sequence distributions from MS spectra remains a formidable challenge, because MS signals are inherently nonquantitative: ionization efficiencies strongly depend on chemical structure. To overcome this limitation, we recently developed a polymer sequencer based on pyrolysis mass spectrometry (pyrolysis-MS).^19^ In this approach, pyrolyzed fragments retain partial sequence information; however, the relative intensities of MS peaks cannot be directly interpreted as sequence occurrence frequencies due to the ionization bias. We addressed this issue by representing the spectra of analyte random copolymers as linear combinations of those from hypothetical sequence-defined polymers, e.g., (XXX)_l_, (XXY)_l_, (XY)_l_, (YYX)_l_, (YYY)_l_ for X/Y copolymers. The mixing ratios of these virtual polymers then represent the analyte’s sequence distribution, enabling quantitative sequence analysis. Although such sequence-defined polymers cannot be synthesized or directly measured, their spectra can instead be virtually generated from data sets of random copolymers with varying compositions using the reference-free quantitative MS (RQMS) algorithm, which employs machine-learning techniques.^20–22^ The only assumption is that the masses of the oligomer fragments preserve short-sequence information, thereby extending applicability to a broader range of monomers compared with NMR. However, this assumption fails for copolymers that (i) undergo side-chain cleavage prior to main-chain scission, converting different monomer units sharing a common backbone into the identical units, or (ii) selectively depolymerize to single-unit monomers rather than oligomers. As acid-catalyzed decomposability is the essential request for resist polymers, their side chains are labile and undergo decomposition at lower temperature than main chain scissions for oligomeric fragmentation.^23^ Furthermore, methacrylic monomers frequently used in ArF and EUV resists are prone to thermal depolymerization. Consequently, chemically amplified resist polymers fall into both categories and thus yield predominantly noninformative fragments, which makes sequence analysis with pyrolysis-MS particularly challenging.

However, even in such copolymers that decompose into fragments whose masses cannot preserve the sequence information, traces of the original sequence should still be preserved in some other form. In this study, we explore the possibility that the instabilities toward depolymerization and side chain cleavages vary depending on the adjacent monomer species, leading to sequence-induced modulations in decomposition temperatures. Pyrolysis-MS gradually heats the analyte polymers while continuously ionizing the evolving gases and collecting spectra at short intervals, resulting in a two-dimensional spectrum with mass (*m*/*z*) and temperature axes, both of which carry rich structural and kinetic information.^24,25^ By focusing on the temperature axis, sequence information encoded in decomposition temperature could be revealed. Here, we address the sequence distribution analysis of representative ArF resist polymers composed of thermally degradable 2-methyl-2-adamantyl methacrylate (M) and relatively stable γ-butyrolactone methacrylate (G),^11^ which generate noninformative fragments due to depolymerization and side chain decomposition.^23^ Throughout the data-driven approach, we elucidated that the side chain stability of M depends on the sequence, following the order MMM > MMG > GMG. This finding was further verified by density functional theory (DFT) calculations, in which we evaluated the ester bond dissociation energies of central M units in trimer models while explicitly considering their tacticity conformations. This integrated approach enables sequence analysis of resist polymers and shows that lability to deprotection is sequence-dependent. Although direct sequence–LER correlation analysis is beyond the scope of this study, the observed sequence-dependent lability clearly highlights its importance for understanding resist performance.

## Experimental Section

### Synthesis of G/M Copolymers

A typical free-radical copolymerization procedure for the M/G copolymers is described below. Degassed M monomer (234 mg, 1.0 mmol) and G monomer (170 mg, 1.0 mmol) were placed in a vial and dissolved in a mixed solvent of 1,4-dioxane (3 mL) and *N*,*N*-dimethylformamide (3 mL). An initiator solution of azobis­(isobutyronitrile) (16.4 mg, 0.1 mmol) dissolved in 0.2 mL of dioxane was then added. Polymerization was carried out under a nitrogen atmosphere at 60 °C for 3 h. The resulting reaction mixture was passed through a 0.2 μm filter to remove the aggregated fraction and then dropped into methanol. The resulting suspension was centrifuged at 11,740*g* (10,000 rpm). Even under this harsh centrifugation condition, the extent of precipitation depended on the M/G monomer feed ratio; only the precipitated fraction was collected and subsequently dried under vacuum at 80 °C to yield the copolymer (*M*_n_ = 21,000; *M*_w_/*M*_n_ = 1.63; determined by gel permeation chromatography using tetrahydrofuran as the eluent and poly­(methyl methacrylate) standards for calibration). The details of all copolymers used in this study are summarized in Table S1.

### Pyrolysis-MS Measurement

Pyrolysis–MS was performed on a mass spectrometer (LCMS-2050; Shimadzu) equipped with a proximity corona discharge ion source^26^ (ChemZo; BioChromato) and a heating block (ionRocket; BioChromato). This ionization method is a type of ambient soft ionization, in which atmospheric moisture is primarily ionized and subsequently ionizes the pyrolyzed gases of the sample without further decomposition. Because the ionization efficiency depends on ambient humidity and can vary by up to a factor of 3 between seasons in Japan, a copper sample pot with two separate wells was specially developed to accommodate both the polymer sample and an internal standard (Figure S1). This design allowed simultaneous measurement and correction of the overall spectral intensity based on the polymer weight *W*_*p*_, internal standard weight *W*_*s*_, and internal standard intensity *I*_*s*_, by applying the correction factor *W*_*s*_/(*W*_*p*_ *I*_*s*_). Such normalization enables fair comparison of spectra and is crucial for constructing reliable data sets over the long-term.^27,28^

In this study, 0.1 mg each of the sample and internal standards were loaded into the pot, and the weights were precisely measured to the microgram level using a microbalance (BM-20; A&D). Biphenyl was chosen as the internal standard because it evaporates around 150–200 °C, prior to polymer decomposition. The two-well design was essential to avoid direct contact between the polymer and the internal standard, ensuring that the internal standard did not affect the thermal behavior of the polymer.

The detector voltage of the MS was set to 1.1 kV, and the corona discharge ionization was set to 2.75 kV. The heating block was programmed to increase from room temperature to 500 °C at a rate of 50 °C/min, reaching the final temperature in 10 min, while MS spectra were acquired over the *m*/*z* range of 50–800 at a rate of 0.2 s/scan. After correcting the spectral intensities by the aforementioned method, 1800 spectra for each sample corresponding to the polymer decomposition region (4–10 min) were extracted and grouped into 36 bins with a temperature resolution of 8.3 °C. The resulting ready-to-use spectral data set is provided as Data S1 in CSV format. These formatted spectra were subsequently analyzed with the reference-free quantitative MS (RQMS) algorithm described in our previous report,^19^ implemented in Python 3.9.12.

### Computational Methods

All computational calculations were performed using the Gaussian 16 Rev. C.01 suite (Gaussian Inc.). Geometry optimizations and frequency calculations were carried out at the (U)­B3LYP/6-31G­(d,p) level of theory.^29–31^ The C­(methyladamantyl)–O (COO) bond dissociation energy (BDE) was evaluated as follows. The stereoregularity relevant to this analysis is triad tacticity. In the polymer chain, the triad under investigation is flanked by polymer segments consisting of M or G monomers. To reduce computational costs, however, the terminal monomer units of the trimer models used for quantum chemical calculations were replaced with methyl groups. Because such simplification could potentially affect the most stable conformation of the trimer, a stereoregular pentamer possessing the same tacticity was first subjected to a conformational search using the Balloon program. Among the obtained conformers, the lowest-energy structure was selected, and the corresponding central triad was assumed to represent the most stable conformation in the polymer chain. Based on this structure, a trimer for geometry optimization was constructed by replacing both terminal monomer units with methyl groups. During the geometry optimization, no rotation occurred around the single bonds that determine the tacticity of the polymer chain. Electronic energies including zero-point energy were obtained from frequency calculations of the optimized trimers, the corresponding oxygen-radical species, and the methyladamantyl radical.

## Results and Discussion

### Loss of Sequence-Informative Oligomer Peaks in Pyrolysis–MS

Pyrolysis-MS directly provides sequence information when thermal decomposition yields oligomeric fragments that retain local monomer connectivity. However, for M/G copolymers, we found that such sequence-informative oligomer peaks are almost absent. To clarify this issue, we first present the raw pyrolysis–MS spectra of a 1:1 random M/G copolymer (Figure 1). This spectrum clearly reveals two major processes: the formation of 2-methyleneadamantane (*m*/*z*: 149 with proton adduction) arising from M side chain cleavage at 200–350 °C, and the generation of depolymerized G monomers (*m*/*z* 171) accompanied by side chain scission (*m*/*z* 85) around 300–450 °C. No significant peaks were observed above *m*/*z* 400, indicating that pyrolysis produced only short fragments no larger than dimers, considering the molecular weights of the monomers (M, 234.1 Da; G, 170.1 Da). In addition, simultaneous side chain scission of both M and G monomers led to conversion into poly­(methacrylic acid) homopolymer. The combined effects of monomer-unit depolymerization and parallel homopolymer formation largely erased sequence information.

**Figure 1:**
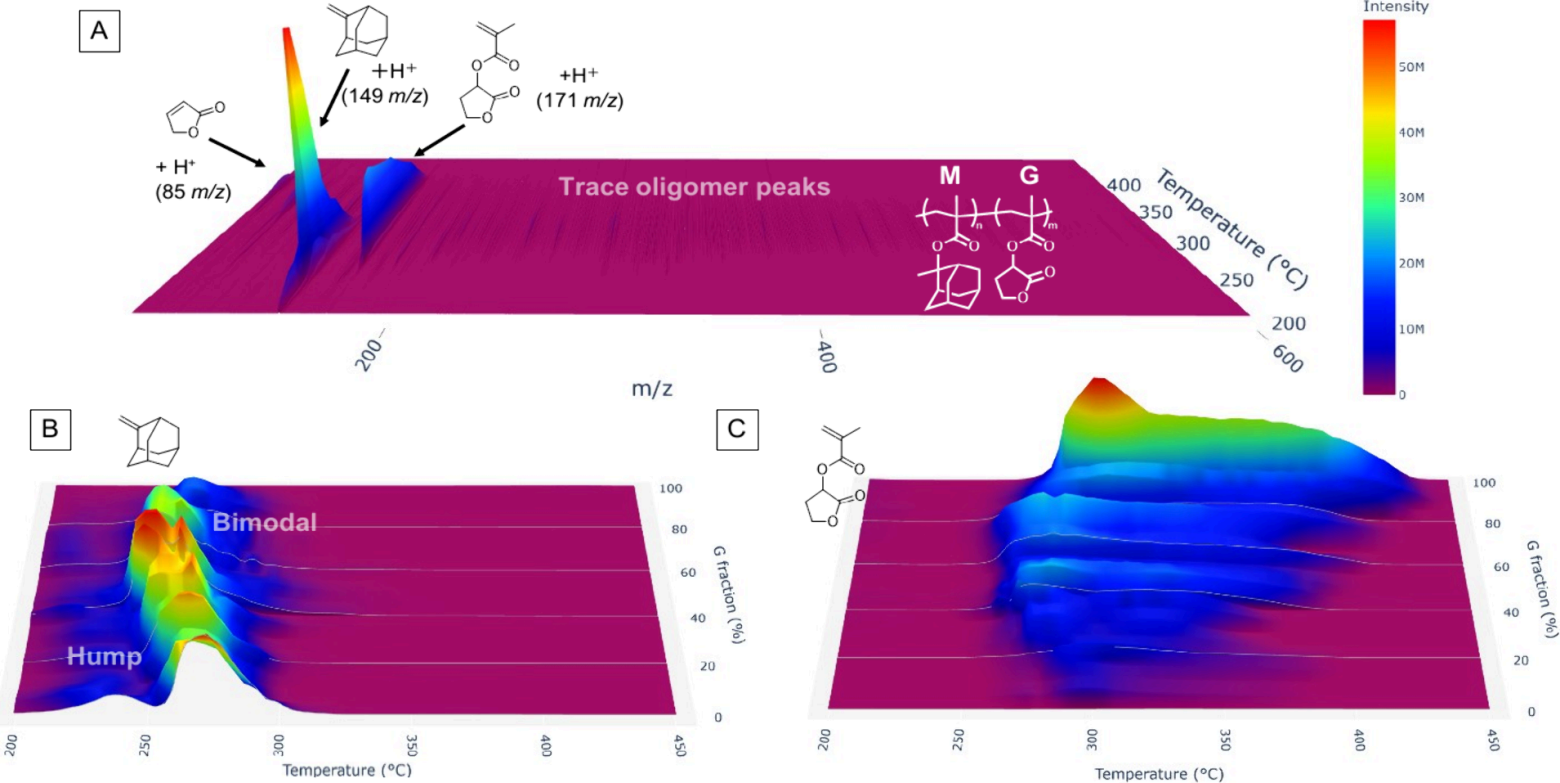
Loss of sequence-informative oligomer peaks in the pyrolysis–MS of M/G copolymers. (A) The *m*/*z*–temperature intensity map of a random copolymer synthesized with a 1:1 monomer feed ratio. (B,C) Temperature-dependent peak intensities of 2-methyleneadamantane (*m*/*z* 149.1) and depolymerized G monomer (*m*/*z* 171.1) in 21 copolymers with various G feed ratios from 0 to 100%. The color scales are identical to that in (A).

Notably, however, the peak intensities of the M side chain fragment and the depolymerized G monomer exhibited distinct temperature dependencies depending on the monomer feed ratio (Figure 1B,C). For the M homopolymer (corresponding to 0% G fraction), the peak assigned to 2-methyleneadamantane reached its maximum intensity around 275 °C, accompanied by a minor hump at lower temperatures (240 °C; Figure 1B). This suggests that side chain instability is also influenced by tacticity. By contrast, in copolymers with higher G fractions, the main peak shifted toward lower temperatures and developed into a bimodal distribution, indicating sequence-dependent modulation of stability. Meanwhile, the peak corresponding to the depolymerized G monomer (*m*/*z* 171.1) also showed pronounced temperature dependence, with depolymerization initiating at the lowest temperatures when the G fraction was approximately 50% (Figure 1C). These results suggest that both side chain and main chain stabilities are strongly influenced by copolymer sequence. Nevertheless, a method to quantitatively determine sequence distribution from the temperature-domain data remains elusive. In the following sections, we first outline how sequencing can be addressed within the framework of compositional analysis using the reference-free quantitative MS (RQMS) algorithm,^19^ and then describe the extensions necessary to incorporate temperature distributions. We then apply the extended RQMS to a data set of 21 M/G copolymers to estimate temperature distributions of fragments derived from sequence-defined polymers, thereby examining how sequence and tacticity affect polymer instability. These conclusions, derived from experimental and data-driven approaches, are further validated using DFT calculations. Finally, we present the mathematical formulation of the extended RQMS that explicitly incorporates temperature distributions.

### Extension of RQMS for Recovering Sequence Information from Temperature Profiles

Figure 2 illustrates the key idea of our approach: the fragment patterns produced by thermal decomposition of a copolymer can be expressed as a linear combination of the patterns from *K*–sequence-defined copolymers, with the mixing fractions (*c*_1_,*c*_2_,...,*c*_*K*_) directly corresponding to the sequence distribution. When fragmentation produces sequence-informative fragments (Figure 2, top), the fragment abundances (FAs) of the sequence defined copolymer can be inferred from the random copolymer data set, thereby allowing determination of the sequence distribution in the analyte.^19^ Importantly, we do not treat all individual triads (2^3^ = 8 in a binary system) as an independent basis set. Instead, we use five sequence-defined copolymers whose triad motifs repeat along the chain (e.g., (XXX)_*l*_, (XXY)_*l*_, (XY)_*l*_, (XYY)_*l*_, (YYY)_*l*_) as the basis set, as schematically shown on the right of Figure 2. A sequence-defined copolymer such as (XXY)_*l*_ corresponds to an infinite repetition −XXY–XXY–XXY–..., and should be distinguished from an isolated XXY trimer. Main-chain scission of (XXY)_*l*_ during pyrolysis generates a characteristic fragment set {XXY, XYX, XX, XY, X, Y} that always appear together with fixed relative intensities and a common temperature profile. Thus, the abundances of all triad, diad, and monomer fragments are determined by the mixing ratios of the underlying sequence-defined copolymers. This representation captures all short-range sequence information while reducing the number of basis elements (from 8 possible triads to 5 sequence-defined copolymers in the binary case), thereby suppressing the combinatorial explosion of basis size for longer sequences or multiple monomer systems.^19^

**Figure 2:**
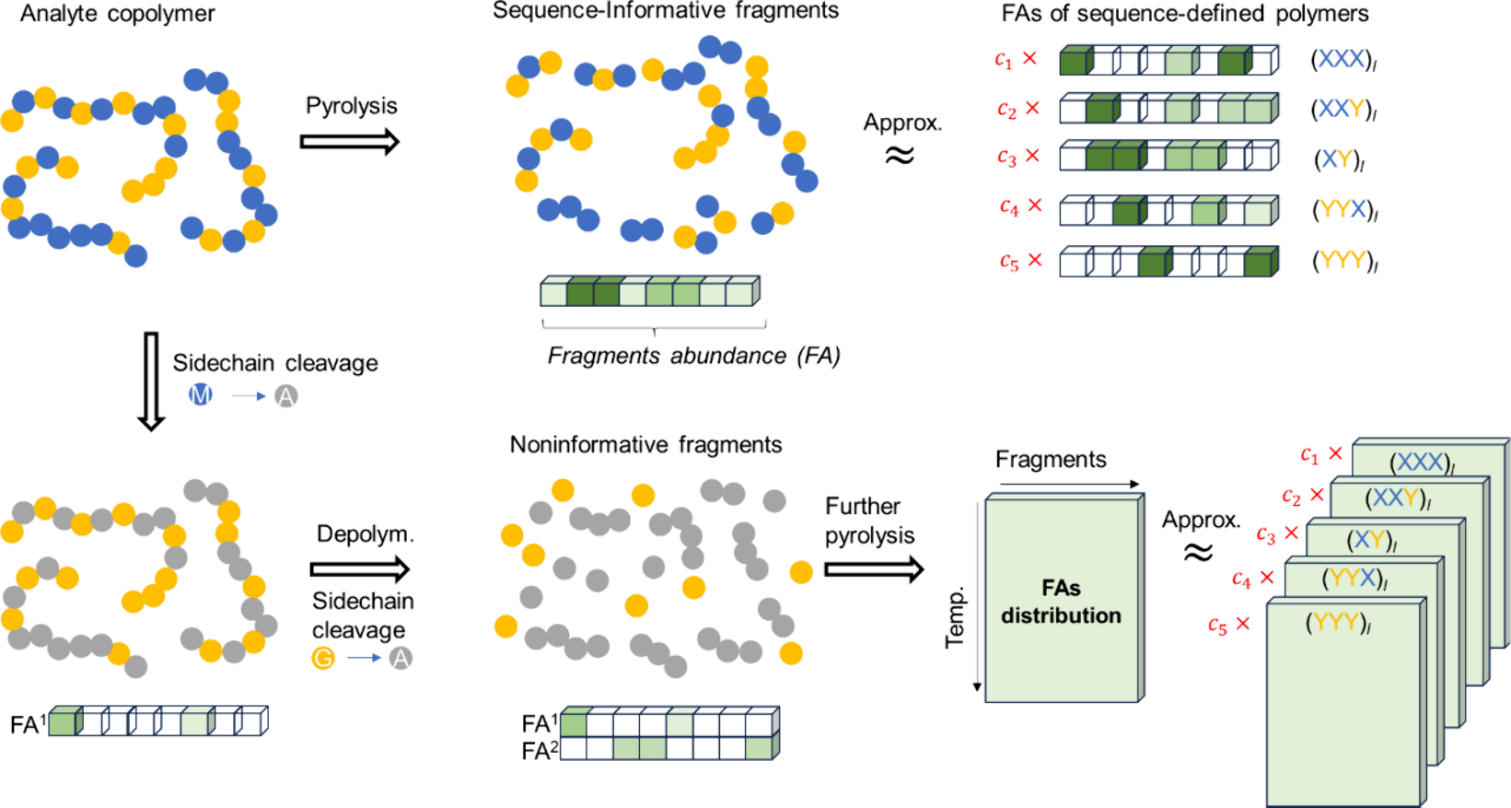
Principle of sequence reconstruction using the extended RQMS framework. (top) Copolymers yield *sequence-informative fragments* enabling reconstruction of sequence distributions from fragment abundances (FAs) using conventional RQMS. (bottom) In M/G copolymers, direct sequence information is lost due to side chain cleavage (M/G → A: methacrylic acid units) and depolymerization, yielding *noninformative fragments.* Nevertheless, sequence-dependent features emerge in the temperature distribution of FAs, thus enabling sequence reconstruction via extended RQMS.

However, in systems where side-chain cleavage and depolymerization dominate (e.g., M/G copolymers; Figure 2, bottom), sequence-informative fragments are no longer available. Both monomer units convert into methacrylic acid (A), while G units preferentially depolymerize into single-unit fragments, leaving little sequence information in the FAs themselves. The key advance of this study is that even in such cases, the temperature distributions of FAs remain sequence-dependent. By extending reference-free quantitative MS (RQMS) to incorporate these temperature distributions, we show that sequence reconstruction is still possible within the same linear-combination framework.

RQMS proceeds in two stages. First, non-negative matrix factorization (NMF)^21^ is applied to the matrix of temperature-resolved spectra and yields a set of fragment spectra that represent recurring substructural motifs across the data set. NMF is well-known as a parts-based, additive decomposition method, and this is particularly well suited to pyrolysis–MS, which does not observe intact polymers but rather the ensemble of “polymer parts” generated by thermal decomposition. In practice, peak intensities that originate from the same polymer substructure tend to increase and decrease in fixed ratios across the data set, and NMF groups such covarying peaks into a single fragment spectrum. Thus, in our terminology a “fragment’’ does not correspond to a single m/z peak, but to a group of peaks whose intensities consistently co-vary across samples, providing a compact and chemically interpretable representation of the complex pyrolysis–MS spectral set. Second, the temperature distributions of FAs, rather than only their integrated abundances, are expressed as linear combinations of those from hypothetical sequence-defined copolymers, with the mixing coefficients directly representing the sequence distributions.

At this stage, highlighting the conceptual differences between conventional kinetic sequence analysis and RQMS-based sequencing is particularly meaningful. Classical copolymerization kinetics can predict long-range sequence tendencies from monomer reactivity ratios,^32^ whereas RQMS directly probes only the short-range sequence. In this sense, the present method provides a compositional analysis of pseudo-repeating units (triads) rather than a reconstruction of long-range sequence tendencies. Nevertheless, because monomer reactivity ratios can, in principle, be inferred from triad compositions,^33^ these techniques are fundamentally connected. The advantages of RQMS become evident when the objective extends from sequence analysis to sequence control: in sequence-control strategies, temperature, solvent, and additives are intentionally adjusted to modulate the monomer reactivity ratios, so approaches that rely on those variable parameters cannot provide an independent verification of the resulting sequence distribution. In contrast, once reference spectra have been inferred for a given set of monomers, RQMS can be applied consistently to copolymers prepared under arbitrary reaction conditions, thereby enabling real-time monitoring of the sequence distributions produced as the monomer reactivity ratios are deliberately controlled.^19^

Note that the relevant technique of pyrolysis gas-chromatography (GC)–MS has advanced considerably, now even capable of resolving tacticity differences in polypropylene based on GC retention time, but it relies on flash pyrolysis and does not retain temperature-resolved fragment formation.^34^ Because our sequencing method critically depends on high-resolution temperature profiles, Py-GC-MS is not suitable for this purpose.

### RQMS-Sequencing for M/G Copolymers

Figure 3A shows the fragment spectra obtained in the first stage of RQMS by NMF, and Figure 3B shows their temperature distributions (numerical data is given in Data S2). Each fragment spectrum was normalized so that the total peak intensity summed to unity before calculating FA distributions. Along the temperature axis, 1,800 spectra were acquired during polymer decomposition (200–500 °C, corresponding to 4–10 min after the onset of heating), which were then divided into 36 bins, giving a temperature resolution of ∼8.3 °C.

**Figure 3:**
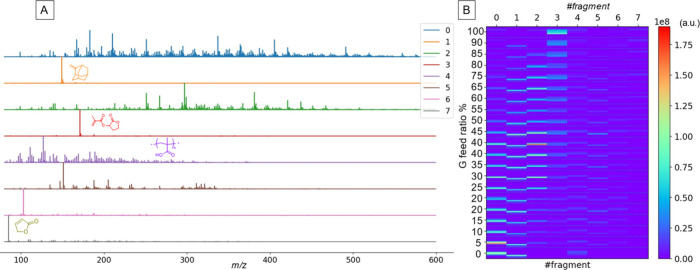
Fragment spectra and their abundances across the data set. (A) Fragment spectra extracted by NMF in the first stage of RQMS. (B) Heatmap showing the distributions of the fragments. The vertical axis corresponds to all spectra acquired for the M/G copolymer data set, ordered by increasing G feed ratio (top to bottom) and, within each sample, subdivided into 36 bins along the temperature axis (200–500 °C, bottom to top; 8.3 °C resolution). Each cell therefore represents the abundance of a given fragment at a specific temperature for a specific copolymer composition. The color scale indicates the abundance of each L1-normalized fragment spectrum in Figure 3A, shown in arbitrary units.

Among the fragment spectra, side chain fragments (spectra 1 and 7) and the depolymerized G monomer (spectrum 3) were assigned based on their corresponding *m*/*z* values. In addition, a complex but reproducible spectrum (spectrum 4), consistently present across all monomer compositions including both M and G homopolymers, was attributed to methacrylic oligomers derived from the common backbone of M and G. The formation of methacrylic oligomers was consistent with the observation of decomposed side chain fragments both from the M and G repeating units.

Other complex spectra, such as spectra 0 and 2, could not be assigned unambiguously but were predominantly observed in M-rich samples (spectrum 0) or at ∼50:50 M/G composition (spectrum 2), suggesting origins from MM and MG diad sequences, respectively. However, unlike previously studied copolymers such as styrene/*n*-butyl acrylate, where fragment peaks corresponding to trimers were clearly observed at masses equal to the sum of three monomer units, no such peaks corresponding to MMM, MMG, or GGM were detected here. Because these masses lie above *m*/*z* ≈ 600, where almost no peaks were observed, we conclude that the FAs themselves do not retain sequence information beyond dimers, and that the temperature distributions of FAs must be utilized to reconstruct triad sequence distributions.

Next, from the fragment distributions of the random copolymers (Figure 3B), we estimated the fragment distributions of five sequence-defined polymers (MMM)_*l*_, (MMG)_*l*_, (MG)_*l*_, (MGG)_*l*_ and (GGG)_*l*_ via the second NMF. Among these, (MMM)_*l*_ and (GGG)_*l*_ correspond to homopolymers that can be experimentally synthesized and measured, leaving (MMG)_*l*_, (MG)_*l*_ and (MGG)_*l*_ to be inferred. For each sample, the FA across *M*-fragments and *N*_*T*_-temperature bins can be represented as a (*N*_*T*_,*M*) matrix. In conventional RQMS analysis, this matrix was integrated along the temperature axis, yielding *M*-dimensional FA vector for each sample while discarding temperature-distribution information. This was appropriate when fragments themselves preserved sequence information, since temperature distributions were unnecessary and could even distort the analysis due to artifacts such as sample loading differences or heat-transfer variations. In contrast, the present study focuses explicitly on temperature distributions. Instead of integration, each (*N*_*T*_,*M*)-matrix was flattened into a *N*_*T*_ *M*-dimensional vector (Data S2), and the goal of the second NMF was to identify five basis vectors that can best reconstruct the FA distributions of all *N*-samples. Importantly, in this (*N*_*T*_,*M*)-matrix representation of FAs the row and column dimensions have fundamentally different meanings. Errors along the fragment axis correspond to assigning intensities to the wrong chemical species, which must be minimized strictly. By contrast, errors along the temperature axis merely reflect differences in the thermal profiles of the same fragment, which can arise from unavoidable experimental factors such as sample placement or loading amount. Consequently, such errors should be treated more leniently. The mathematical procedure we developed to incorporate this distinction is described later; here we first present the results.

Figure 4A,B show the inferred temperature distributions of key fragments, 2-methyleneadamantane and depolymerization G monomer, in the sequence-defined polymers. The full set of temperature distributions for all eight fragments extracted in Figure 3 is provided in Figure S2, and these were used to estimate the sequence distributions of each sample shown in Figure 4C (numerical data is given in Table S2). The inference was carried out such that the fragment distributions of all samples in the data set were well approximated by the linear combinations of inferred distributions (see Figure S3–S4; the detailed mathematical procedure is described later). To examine the accuracy of this result, we compared the G-monomer composition back-calculated from the triad distributions with that determined by ^1^H NMR (Figure 4D). The two methods agree well across most of the composition range. A small but systematic deviation appears in the low-G limit (G feed <0.2), where the RQMS-derived values overestimate the incorporation of G. In this region, the polymer should consist almost entirely of MMM triads, with only a minor fraction of MMG/MGM; therefore, the apparent overestimation of G corresponds to an underestimation of (MMM)_*l*_ and/or an overestimation of (MMG)_*l*_. This bias may be attributed to the lower molecular weight of M-homopolymer compared with M/G copolymers (Table S1). Although individual terminal fragments cannot be explicitly assigned within the complex M-homopolymer spectrum, the low molecular weight of M-homopolymer implies that terminal structures contribute substantially to the (MMM)_*l*_ spectrum. In contrast, M/G copolymers possess higher molecular weights, and the contribution of terminal fragments should be much smaller. Consequently, when decomposing copolymer spectra into (MMM)_*l*_ and (MMG)_*l*_, the less significant contributions of terminus in copolymers cause the (MMM)_*l*_ component to be underestimated, which in turn leads to a overestimation of the G-monomer content in the M-rich region. These observations highlight an important practical guideline for constructing reliable RQMS data sets: to minimize terminal-structure contributions, particularly for monomers with large molecular weights, at least *M*_n_ > 10,000 is desirable. Furthermore, stopping the polymerization at relatively low conversion is helpful for obtaining higher-quality data sets, because high-conversion samples inevitably broaden sequence distribution, which complicate the mathematical estimation of virtual reference spectra corresponding to sequence-defined polymers.

**Figure 4:**
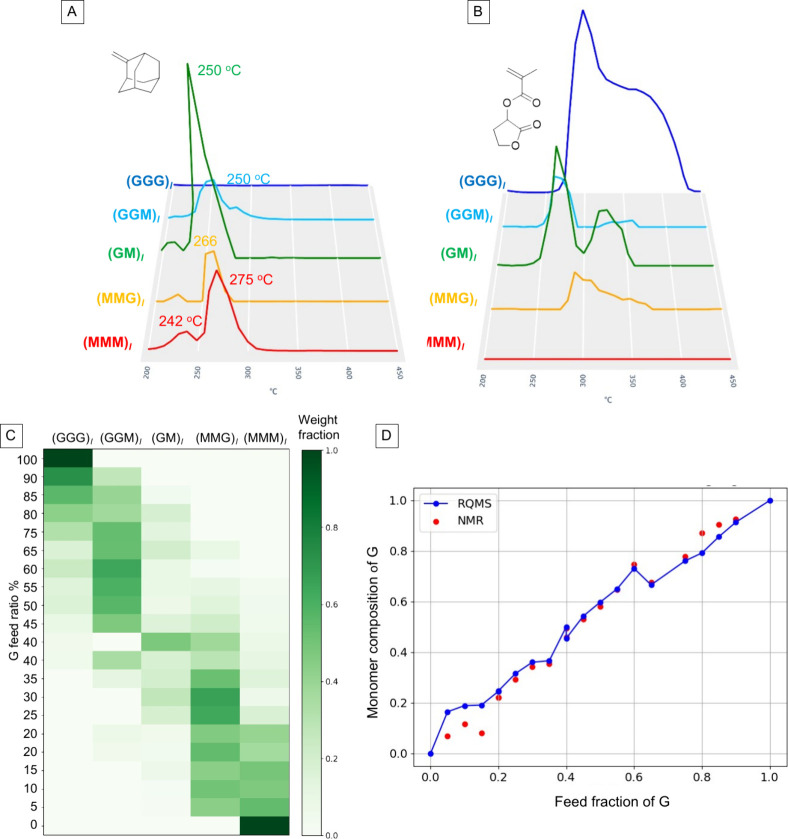
RQMS sequencing for M/G copolymers. (A,B) Inferred temperature distributions of fragment abundances (FA) for fragments 1 and 3 in Figure 3A. The vertical axis represents the abundance of each L1-normalized fragment spectrum, shown in arbitrary units but directly comparable among the sequence-defined copolymers. (C) Sequence distributions of random copolymers, obtained by decomposing the full set of fragment temperature distributions for each sample into five sequence-defined copolymers (MMM)_*l*_, (MMG)_*l*_, (MG)_*l*_, (MGG)_*l*_, and (GGG)_*l*_. (A) and (B) illustrate representative fragments, whereas (C) summarizes the overall sequencing results based on all fragments (see numerical data in Table S2). (D) G-monomer composition in the isolated copolymers plotted as a function of the G feed ratio. Blue line: back-calculation from the RQMS-derived triad distributions. Red dots: ^1^H NMR determination.

This sequence analysis not only visualizes the sequence composition explicitly for each sample but also reveals how the sequence affects the decomposition temperature. In particular, for M monomers, the side chain functions as a protecting group in resist polymers and is converted to a carboxylic acid by acid-catalyzed deprotection, thereby rendering the polymer soluble in alkaline developer to form patterns. If the instability of this protecting group depends on the sequence, then sequence inhomogeneity will translate into spatial variations in the extent of deprotection under identical exposure and postexposure processing conditions. Such spatial variations in the degree of deprotection, and the associated chemical-reaction nonuniformity, have been suggested both in early studies on chemically amplified resists and,^35^ more recently, in stochastic analyses of EUV resists to be important sources of chemical noise and LER.^36^ Clarifying this sequence–instability relationship is therefore key to understanding how sequence design might ultimately contribute to LER control.

Figure 4A shows the decomposition temperature of M-side chains decreases in the order (MMM)_*l*_ → (MMG)_*l*_ → (MG)_*l*_, with (MGG)_*l*_ and (MG)_*l*_ exhibiting nearly identical peak temperatures. This indicates that within M-side chain stability is affected by the adjacent monomers: most stable when flanked by two M units, while substitution by one or two G units leads to progressive destabilization. Notably, (MG)_*l*_, despite containing only half as many M units as (MMM)_*l*_, exhibited the strongest signal, indicating that when flanked by two G units, the M side chain becomes more labile and undergoes selective side-chain cleavage. Interestingly, however, the M homopolymer showed not only a major decomposition peak at 275 °C but also a minor peak at 242 °C, suggesting that tacticity may also affect the side chain stability.

To investigate this, we analyzed the tacticity of the M homopolymer by ^13^C NMR, focusing on the quaternary carbon of the adamantyl group (Figure S5). The peak assignment was made in analogy to poly­(adamantyl methacrylate), and the tacticity was determined to be *mm*/*mr*/*rr* = 57/38/5. This tacticity was markedly different from that of poly­(adamantyl methacrylate) prepared by radical polymerization at 60 °C (*mm*/*mr*/*rr* = 3/30/67),^37^ indicating that the methyl group at the 2-position of adamantane strongly influences stereoregularity. For extremely bulky side chains, it has been reported that the *mm* configuration induces a helical backbone conformation that orients the bulky substituents outward;^38,39^ a similar conformational preference may explain the unusual tacticity observed here.

Nevertheless, the M homopolymer exhibited only two distinct decomposition peaks, making it difficult to assign them to the *mm*/*mr*/*rr* three conformations. We therefore employed a computational approach to evaluate the effect of tacticity on the bond dissociation energy (BDE) of the 2-methyladamantyl ester in M-centered triads. Figure 5 outlines the modeling procedure for triad sequence and tacticity analysis, and the detailed computational protocol is described in the Experimental Section. The calculated BDEs were 298 kJ/mol for the *mm* configuration and 297 kJ/mol for *mr*, whereas the *rr* configuration gave a significantly lower value of 285 kJ/mol. These results indicate that the minor peak at 242 °C corresponds to the *rr* configuration, while the major peak at 275 °C arises from the combined contributions of the *mr* and *mm* configurations. This interpretation is fully consistent with the experimental triad fractions (*mm*+*mr*/*rr* ∼ 95/5), which account for the small 2-methyleneadamantane peak at lower temperature and the dominant peak at higher temperature.

**Figure 5:**
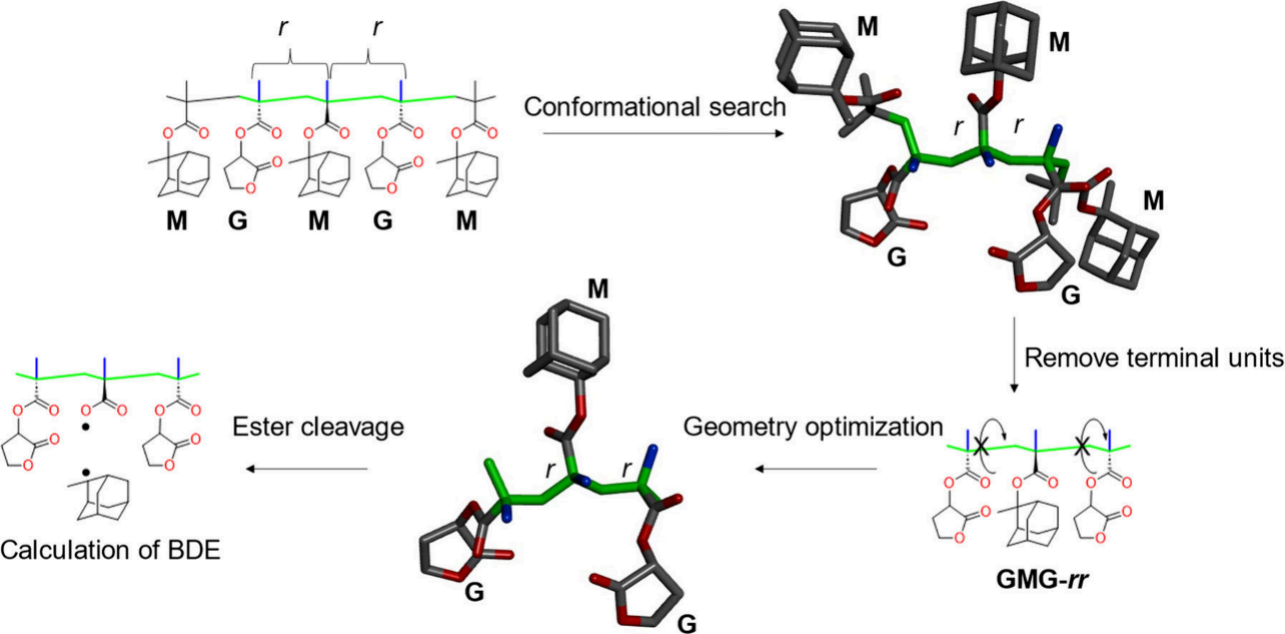
DFT calculation of the bond dissociation energy (BDE) of a 2-methyladamantyl ester in the central M units of GMG-*rr* triads. Conformational searches were carried out on pentamer models in which terminal units rotated freely, leaving *r*/*m* stereodescriptors at the chain ends undefined. Geometry optimizations were subsequently performed on clipped trimer models, preserving central stereochemistry. Green indicates main-chain carbons, blue indicates α-methyl groups, and red indicates oxygen atoms. Hydrogen atoms are omitted for clarity.

Next, we further investigated sequence effect. For the GMG triad, the calculated BDEs were nearly identical across configurations, *rr*, 286 kJ/mol; *rm*, 289 kJ/mol; *mm*, 291 kJ/mol, indicating that the sterically less bulky G units reduce conformational influence. The overall BDE of GMG thus laid between MMM-*rr* and MMM-*rm*/*mm*, consistent with the decomposition temperature profile of the alternative sequence (MG)_*l*_, which showed a strong peak at 250 °C. These results provide a coherent explanation for the temperature distribution of 2-methyleneadamantane arising from M-side chain cleavage in sequence-defined polymers predicted by RQMS, thereby validating the predictions in Figure 4 and supporting the reliability of the sequence distributions inferred for random copolymers.

### Temperature Distribution Inference of FAs in Sequence-Defined Polymers

We extended the RQMS framework to infer the temperature distributions of fragment abundances (FAs) in sequence-defined polymers. Our notation follows the standard conventions adopted in signal processing (see Mathematical notations in Supporting Information). Here, a MS spectrum is represented as a *D*-dimensional row vector containing the non-negative signal intensities across *D*-channels. A pyrolysis-MS spectrum with *N*_*T*_ temperature bands is thus expressed as a two-dimensional (*N*_*T*_,*D*)-matrix. A spectral data set ***X*** consisting of *N*-samples is represented as a non-negative (*N*_*T*_ *N*,*D*)-matrix and here noted as $X\in {R_{+}}^{N_{T}N\times D}$ . The spectrum with *m*/*z* and temperature axes of *n*-th sample is stored from row *N*_*T*_ *n* to *N*_*T*_(*n*+1)-1 in ***X***.

In the first step of RQMS, NMF was applied to ***X***, yielding *M*-fragment spectra $S\in {R_{+}}^{M\times D}$ and fragment abundances $A\in {R_{+}}^{N_{T}N\times M}$ . The block of rows corresponding to the *n*th sample, denoted as ***A*** ^(*n*)^, contains the temperature distribution of FA for that sample. In conventional RQMS, this block was integrated along temperature axis, yielding *M*-dimensional vector corresponding to Figure 2 (top). To fully utilize the temperature information, we instead column-wisely vectorize ***A*** ^(*n*)^ to obtain $a_{n}\equiv vec\left(A^{\left(n\right)}\right)\in {R_{+}}^{N_{T}M}$ . Our objective is to determine the basis vectors $b_{k}\in {R_{+}}^{N_{T}M}\left(k=1,2,...K\right)$ and mixing fractions ***c***_*n*_ = (*c*_*n*1_,*c*_*n*2_,...,*c*_*nK*_) that best approximate each ***a***_*n*_: $a_{n}\approx \overset{K}{\underset{k=1}{\sum }}c_{nk}b_{k}$ . In matrix form, this can be written simply as ***A*** ~≈ CB, where $Ã\in {R_{+}}^{N\times N_{T}M}$ is constructed by stacking ***a***_*n*_^*T*^(*n* = 1,2,...*N*), $C\in {R_{+}}^{N\times K}$ denotes the mixing fractions and $B\in {R_{+}}^{K\times N_{T}M}$ represents temperature distribution of FAs in the references, i.e., sequence-defined polymers.

Importantly, NMF basis are nonorthogonal coordinates. Moreover, when a given fragment shows comparable intensity across adjacent temperature bands, it is not always clear whether this reflects genuine differences in thermal stability arising from distinct substructures or microenvironments, or merely experimental artifacts such as slight variations in sample loading or positioning within the pyrolysis pot that affect heat transfer. Consequently, the abundance of a fragment in one temperature band should be, to some extent, represented by the same fragment in neighboring bands. Thus, $a_{n}\in {R_{+}}^{N_{T}M}$ should not be regarded as a vector in an orthogonal coordinate system; instead, the approximation ***A*** ~≈ CB must be evaluated using a Riemannian distance that accounts for the degree of nonorthogonality between dimensions.^40^ To this end, we introduce a Gram matrix $G\in R^{N_{T}M\times N_{T}M}$ and define the approximation error as

$$D_{G}\left(Ã|CB\right)\equiv Tr\left[\left(Ã-CB\right)G{\left(Ã-CB\right)}^{T}\right]$$

Here, the nonorthogonality of the NMF bases is represented by the cosine similarity between fragment spectra, $G_{S}={SS}^{T}\in R^{M\times M}$ . Furthermore, we introduce temperature-axis nonorthogonality using a Gaussian kernel with one standard deviation corresponding to a single temperature bin (8.3 °C; equivalent to 10 s in heating time). Specifically,

$${G_{T}}_{i,j}=\exp\left(-\frac{{\left|i-j\right|}^{2}}{2}\right),G_{T}\in R^{N_{T}\times N_{T}}$$

Finally, by setting ***G*** = ***G***_*s*_⊗ ***G***_*T*_, we simultaneously account for the nonorthogonality of the NMF fragment bases and the uncertainty along the temperature axis, thereby minimizing the approximation error in this generalized coordinate system. Except for this extension of the Gram matrix, all other procedures followed the algorithm described in our previous report with the given parameters in Table S3.^19^

## Conclusions

Polymer sequencing based on pyrolysis-MS has emerged as a next-generation alternative to NMR, offering broad applicability to diverse monomers and rapid measurement. Yet its use has been confined to copolymers that generate sequence-informative fragments. By contrast, methacrylate copolymers, technologically important as resist materials, undergo side-chain cleavage and selective depolymerization, producing mostly noninformative fragments and preventing sequence analysis. In this study, we found that these instabilities themselves are sequence-dependent. By exploiting temperature distributions of fragments, we successfully achieved sequencing of resist polymers. Notably, the predicted temperature distributions of protective-group fragments from sequence-defined polymers, inferred using machine learning, were validated by DFT calculations. The deprotection sensitivities of the M side chain follow the order: MMM-*rr* > MMG > GMG > MMM-*rm*/*mm*. These results clearly demonstrate that sequence directly affects deprotection sensitivity, underscoring the importance of advancing sequence–LER correlation analysis in resist design. As a proof of concept, this study focused on the fundamental binary methacrylate copolymers. Finally, we outline how the present framework could be applied to industrially relevant resist polymers, where a third monomer such as 3-hydroxyadamantyl methacrylate is often introduced to improve substrate wetting.^5^ Although this monomer shares the same adamantyl side chain as the acid-labile adamantyl methacrylates, it is not an unstable tertiary ester and is therefore more likely to yield sequence-informative fragments upon pyrolysis. In this sense, extending the present RQMS framework to ArF-type ternary methacrylate systems should be feasible in principle, provided that the data set is sufficiently enriched to cover the additional triads. By contrast, state-of-the-art EUV resists incorporate elements with large photoabsorption cross sections, such as iodine,^41^ together with ionic photoacid-generator-bound monomers to improve LER.^1^ Both features tend to reduce volatility, making it difficult to generate sequence-informative fragments. Nevertheless, even in such systems, the present strategy, relying on temperature-resolved side-chain scission profiles rather than complete volatilization of the backbone, may provide a useful route to extract sequence information and thereby enable quantitative evaluation of how sequence distributions impact the performance of state-of-the-art resists.

## Supplementary Material





## Notes

21 copolymers used in this study, RQMS parameters, and Figures 1–5 (PDF)
the ready-to-use spectral set, processed numerical data for ***A***, mathematical notation (ZIP)
