# Supplementary material for: Reference-Free Quantitative Mass Spectrometry Enables Sequencing of Resist Copolymers and Reveals Sequence-Dependent Deprotection Sensitivity
Source: Macromolecules. 2026 Jan 19;59(3):1640–9. doi: 10.1021/acs.macromol.5c03032 (PMC12895505; doi:10.1021/acs.macromol.5c03032)
Supplement: Supplementary file 1 [file ma5c03032_si_001.pdf]

Support Information for

Reference-Free Quantitative Mass Spectrometry Enables Sequencing of Resist Copolymers and Reveals Sequence-Dependent Deprotection Sensitivity

Yusuke Hibi*[1], Yasuyuki Nakamura[1], Shiho Uesaka[1], and Masanobu Naito[1]

[1]Data-driven Polymer Design Group, Research Center for Macromolecules and Biomaterials, National Institute for Materials Science (NIMS); 1-2-1, Sengen, Tsukuba, Ibaraki 305-0047, Japan.

*Corresponding author. Email: hibi.yusuke@nims.go.jp

This PDF file includes:

Methods and Materials

Supplementary Figures

Supplementary Tables

Captions for Supplementary Data

Other Supplementary Materials for this manuscript include the following:

Data S1-2 are attached in csv format.

Methods and materials

Mathematical notations

Non-negative M-dimensional column and row vectors are denoted by $\boldsymbol{a} \in \mathbb{R}_+{}^M$ and $\boldsymbol{a}^T \in \mathbb{R}_+{}^M$ respectively. An (N, M)-dimensional matrix is denoted by $\boldsymbol{A} \in \mathbb{R}_+{}^{N\times M}$. For a square matrix $\boldsymbol{X}$, $Tr(\boldsymbol{X})$ represents the trace of $\boldsymbol{X}$. The operator $vec(\boldsymbol{X})$ denotes column-wise vectorization, stacking the columns of $\boldsymbol{X}$ into a single vector. The Kronecker product of two matrices $\boldsymbol{X}$ and $\boldsymbol{Y}$ is written as $\boldsymbol{X} \otimes \boldsymbol{Y}$.

Materials

High-purity chemicals were purchased from Tokyo Chemical Industry and used without further purification.

Supplementary Figures and Tables

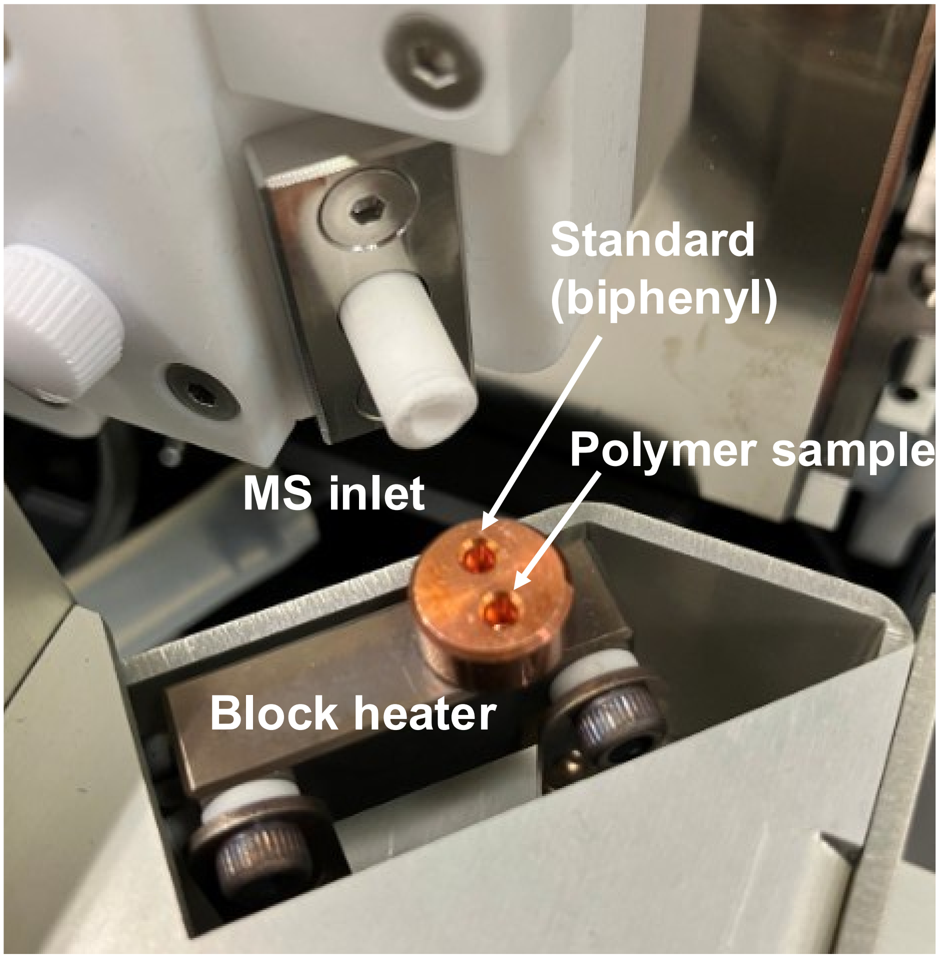

Fig. S1. A specially designed copper sample pot with two separate wells, allowing simultaneous loading of a polymer sample and an internal standard (biphenyl), positioned directly below the MS inlet and heated by a block heater. The MS inlet continuously aspirates at 8 mL/min, so that the evolved gases are immediately drawn in and ionized by the proximal corona discharge (2.75 kV), before being introduced into the MS vacuum chamber with minimal secondary reactions.

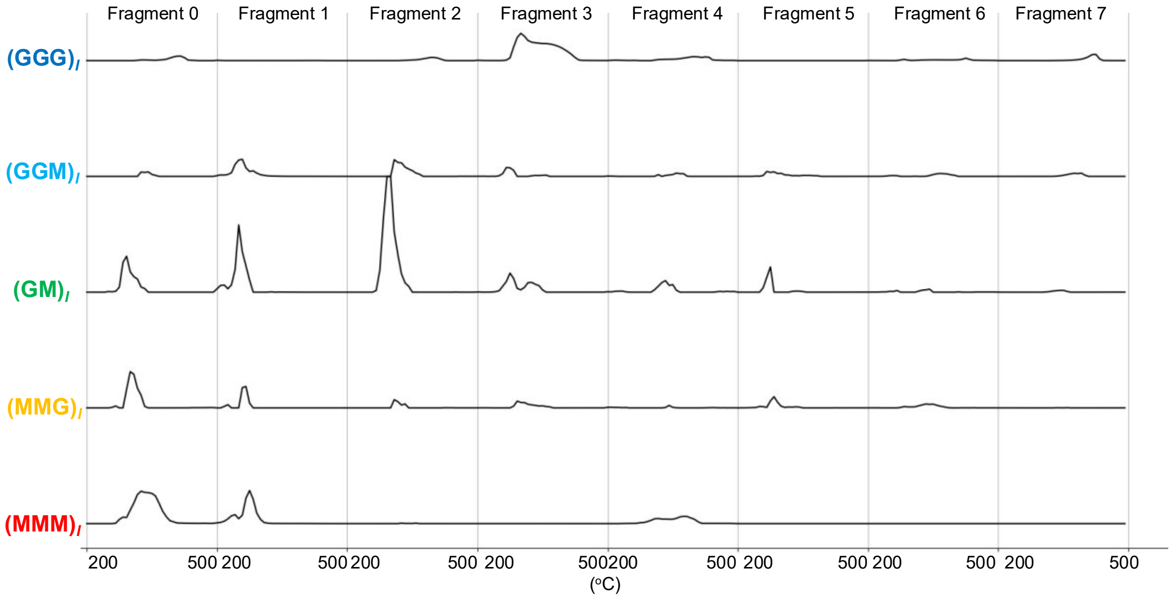

Fig. S2. Temperature distributions of fragment abundances (FA) for all basis fragments in the five sequence-defined copolymers. For each basis fragment (Fragment 0–7, corresponding to the spectra extracted in Fig. 3A), FA profiles were calculated by dividing the thermal decomposition region (200–500 °C) into 36 bins (~8.3 °C resolution). The vertical axis represents fragment abundance, normalized so that intensities are directly comparable among the sequence-defined copolymers. Representative results for fragments 1 and 3 are highlighted in the main text (Fig. 4A-B).

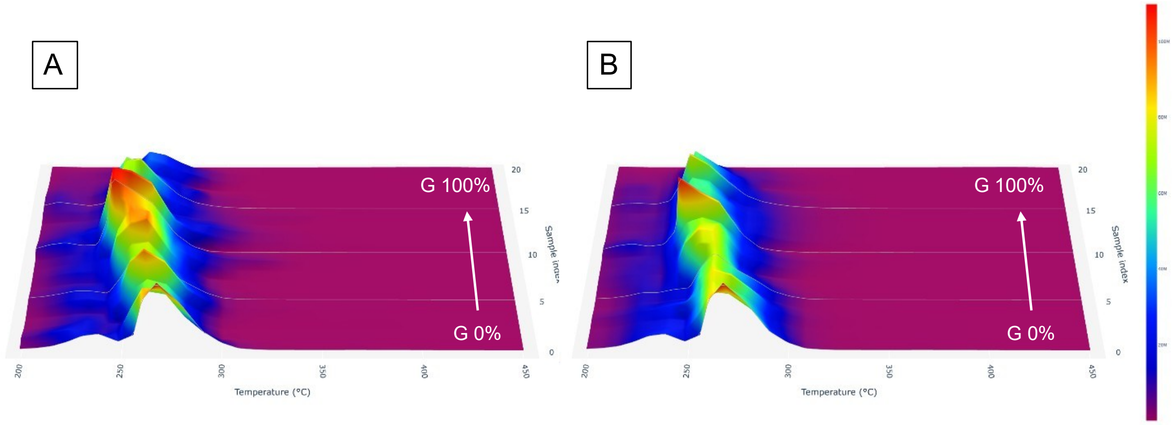

Fig. S3. Temperature distribution of fragment 1 (2-methyleneadamantane). (A) Experimental FA distributions across the M/G copolymer dataset. (B) Reconstructed FA distributions obtained from the inferred contributions of sequence-defined copolymers (corresponding to Fig. 4A).

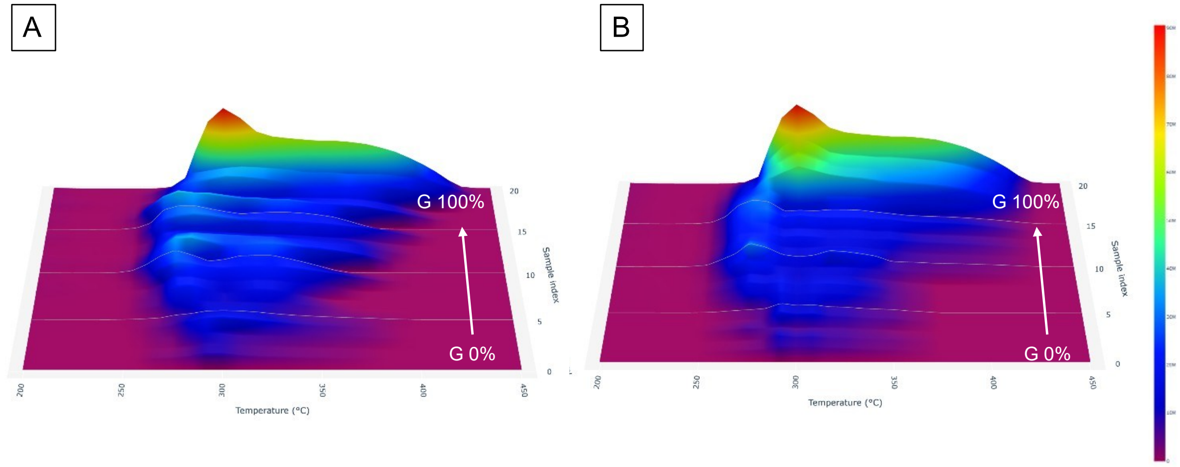

Fig. S4. Temperature distribution of fragment 3 (depolymerized G monomers). (A) Experimental FA distributions across the M/G copolymer dataset. (B) Reconstructed FA distributions obtained from the inferred contributions of sequence-defined copolymers (corresponding to Fig. 4B).

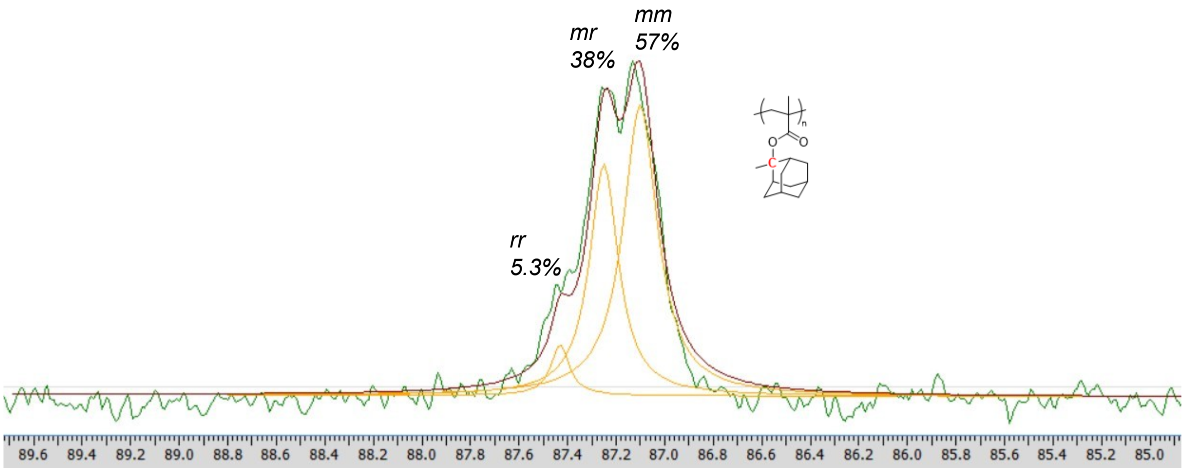

Fig. S5. ^{13}C NMR spectrum of the quaternary carbon in poly(2-methyladamantyl methacrylate). The peak shows a trimodal pattern, which can be assigned to *rr*, *mr*, and *mm* configurations, in the same manner as reported previously for poly(adamantyl methacrylate). The spectrum was measured in $CDCl_3$ (20 wt%) with JEOL ECS-400 at 55 ºC.

Table S1. Copolymers information used in this study.

Sample code	G feed fraction	Reaction Time[h]	Weight-based polymer yield [%]	GPC data [a]		Monomer [b] composition (G fraction)
				M_n	M_w/M_n	
G00M100	0	3	8	7900	1.50	0
G05M95	0.05	3	13	10000	1.53	0.070
G10M90	0.1	3	15	10300	1.57	0.12
G15M85	0.15	3	14	11900	1.63	0.082
G20M80_1h	0.2	1	11	12100	1.67	0.22
G20M80	0.2	3	18	11100	1.69	0.22
G25M75	0.25	3	15	13200	1.63	0.29
G30M70	0.3	3	17	15000	1.59	0.34
G35M65	0.35	3	15	16600	1.57	0.35
G40M60	0.4	3	17	18100	1.60	0.49
G40M60_1h	0.4	1	7	18500	1.65	0.46
G45M55	0.45	3	16	18800	1.63	0.53
G50M50	0.5	3	18	21000	1.63	0.58
G55M45	0.55	3	18	21600	1.63	0.64
G60M40	0.6	3	10	23400	1.62	0.74
G65M35	0.65	3	18	31500	1.95	0.67
G75M25	0.75	3	32	32700	2.00	0.78
G80M20	0.8	3	31	34700	2.00	0.87
G85M15	0.85	3	31	45400	2.22	0.91
G90M10	0.9	3	43	52300	2.16	0.93
G100M00	1	3	50	59000	2.10	1

(a) THF was used as the eluent with PMMA standards for copolymers with a G feed ratio < 65%. For G-rich samples, DMF containing 0.1 wt% LiBr was used as the eluent with PMMA standards because of their low solubility in THF.

(b) Monomer compositions were determined by ^{1}H NMR from the integral ratio of the three lactone-derived protons (α-CH and O-CH_2) to the aliphatic protons.

Table S2. Numerical data of Fig. 4C.

Sample code	Weight fractions of sequence-defined copolymers				
	$(GGG)_l$	$(GGM)_l$	$(MG)_l$	$(MMG)_l$	$(MMM)_l$
G00M100	0.000	0.000	0.000	0.000	1.000
G05M95	0.000	0.000	0.016	0.447	0.537
G10M90	0.000	0.000	0.022	0.511	0.467
G15M85	0.000	0.000	0.067	0.446	0.487
G20M80_1h	0.000	0.053	0.042	0.538	0.367
G20M80	0.000	0.081	0.055	0.458	0.406
G25M75	0.000	0.000	0.196	0.631	0.173
G30M70	0.000	0.000	0.268	0.665	0.067
G35M65	0.000	0.120	0.202	0.529	0.149
G40M60	0.054	0.358	0.184	0.294	0.110
G40M60_1h	0.045	0.000	0.486[a]	0.386	0.084
G45M55	0.097	0.471	0.159	0.217	0.056
G50M50	0.165	0.566	0.074	0.145	0.049
G55M45	0.152	0.598	0.096	0.116	0.038
G60M40	0.241	0.640	0.093	0.025	0.000
G65M35	0.165	0.534	0.202	0.099	0.000
G75M25	0.320	0.543	0.136	0.000	0.000
G80M20	0.424	0.393	0.183	0.000	0.000
G85M15	0.559	0.403	0.038	0.000	0.000
G90M10	0.724	0.273	0.003	0.000	0.000
G100M00	1.000	0.000	0.000	0.000	0.000

(a) The relatively high $(MG)_l$ content may be attributable to stopping the polymerization at an early stage, where the instantaneous monomer composition remained close to the feed and thus afforded higher intrinsic sequence purity. In addition, methanol precipitation at low conversion led to partial polymer loss, which can introduce a mild fractionation effect. The combined influence of these factors can enrich specific sequence populations.

Table S3. RQMS parameters used in this study.[1]

Sample N Temperature Bins N_T	First NMF				Second NMF		
	w_o	Merging threshold	Initial M	iteration	K	$\alpha = \beta$	ρ
N=21, N_T=36	0.2	0.95	20	3000	5	0.05	1.5

The parameters used in the first NMF, which consolidates peaks into basis spectra, are defined as follows:

w_o: Degree of orthogonality imposed among basis spectra. A higher value of w_o leads to fewer shared peaks across spectra. w_o ranges between 0 (no constraint) and 1 (maximal orthogonality constraint).

Merging threshold and initial M: These are linked to the automatic relevance determination (ARD) procedure. The number of initial bases is intentionally set larger than needed, and redundant bases are subsequently pruned by merging once their cosine similarity exceeds the preset merging threshold.

The second NMF, which yields the final compositional outputs, uses the following parameters:

K: Number of underlying components in the system.

α: Regularization parameter that enforces the minimum-volume constraint on the simplex defined by the reference spectra. Increasing α contracts the simplex.

β: Regularization parameter regulating orthogonality among reference spectra. Increasing β: expands the simplex.

ρ: Parameter adjusting sensitivity to outliers, typically chosen between 0.5 and 2. A larger ρ improves responsiveness to data variations but reduces robustness against outliers.

Caption for Data S1.

Intensity-corrected spectra, summarized into 36 temperature bins (200–500 °C) from 1,800 spectra per sample, corresponding to $\boldsymbol{X}$.

Caption for Data S2.

Temperature distributions of fragment abundances, corresponding to the numerical data of matrix $\widetilde{\boldsymbol{A}}$.

Reference

(1) Hibi, Y.; Uesaka, S.; Naito, M. A Data-Driven Sequencer That Unveils Latent "Codons" in Synthetic Copolymers. *Chem. Sci.* **2023**, *14* (21), 5619-5626.
